# Antarctic lake viromes reveal potential virus associated influences on nutrient cycling in ice-covered lakes

**DOI:** 10.3389/fmicb.2024.1422941

**Published:** 2024-09-10

**Authors:** David Robinson, Rachael M. Morgan-Kiss, Zhong Wang, Cristina Takacs-Vesbach

**Affiliations:** ^1^Department of Biology, University of New Mexico, Albuquerque, NM, United States; ^2^Department of Microbiology, Miami University, Oxford, OH, United States; ^3^Department of Energy Joint Genome Institute, Lawrence Berkeley National Laboratory, Berkeley, CA, United States; ^4^Environmental Genomics and Systems Biology Division, Lawrence Berkeley National Laboratory, Berkeley, CA, United States; ^5^School of Natural Sciences, University of California, Merced, Merced, CA, United States

**Keywords:** virus, bacteria, metagenomics, Antarctica, limnology

## Abstract

The McMurdo Dry Valleys (MDVs) of Antarctica are a mosaic of extreme habitats which are dominated by microbial life. The MDVs include glacial melt holes, streams, lakes, and soils, which are interconnected through the transfer of energy and flux of inorganic and organic material via wind and hydrology. For the first time, we provide new data on the viral community structure and function in the MDVs through metagenomics of the planktonic and benthic mat communities of Lakes Bonney and Fryxell. Viral taxonomic diversity was compared across lakes and ecological function was investigated by characterizing auxiliary metabolic genes (AMGs) and predicting viral hosts. Our data suggest that viral communities differed between the lakes and among sites: these differences were connected to microbial host communities. AMGs were associated with the potential augmentation of multiple biogeochemical processes in host, most notably with phosphorus acquisition, organic nitrogen acquisition, sulfur oxidation, and photosynthesis. Viral genome abundances containing AMGs differed between the lakes and microbial mats, indicating site specialization. Using procrustes analysis, we also identified significant coupling between viral and bacterial communities (*p* = 0.001). Finally, host predictions indicate viral host preference among the assembled viromes. Collectively, our data show that: (i) viruses are uniquely distributed through the McMurdo Dry Valley lakes, (ii) their AMGs can contribute to overcoming host nutrient limitation and, (iii) viral and bacterial MDV communities are tightly coupled.

## 1 Introduction

Viruses play significant ecological roles in aquatic systems, specifically through host cell lysis, recycling of nutrients, and augmentation of metabolism during infection (López-Bueno et al., 2009; Aguirre de Cárcer et al., 2015; Coutinho et al., 2017; Warwick-Dugdale et al., 2019). In marine systems, viruses infect and kill microbial hosts including bacteria, archaea, cyanobacteria, protists, and fungi, and are estimated to release 10^8^–10^9^ tons of carbon per day (Suttle, 2005; Coutinho et al., 2017; Yau and Seth-Pasricha, 2019). During infection, viruses can also modulate host cell metabolism by expressing auxiliary metabolic genes (AMGs) (Breitbart et al., 2007; Sime-Ngando, 2014; Warwick-Dugdale et al., 2019). Polar ecosystems are classically truncation food webs, meaning they are missing macrofauna grazing that is classically found in temperate ecosystems. Due to this truncation polar ecosystems are microbially dominated, and viruses in particular, likely play outsized ecological roles in comparison to more complex food webs (Säwström et al., 2008; Laybourn-Parry, 2009; Yau and Seth-Pasricha, 2019).

The lakes of the MDVs of Antarctica are microbially dominated systems due to the low temperature, wide salinity gradients, and the surrounding arid environment (Fountain et al., 1999; Doran et al., 2002). In the MDVs there are no plants or animals, allowing microbial interactions to be more directly assessed (Priscu et al., 1999). The lakes of the MDVs have unique bacterial and eukaryal communities throughout their water columns, owing to distinct geochemistry between and stratification within the lakes (Bowman et al., 2016; Kwon et al., 2017; Li and Morgan-Kiss, 2019). MDV microbial communities have been studied since the 1970s and in recent years high throughput sequencing of 16S rRNA and 18S rRNA genes has led to a deeper understanding of the microbial food web (Bowman et al., 2016; Kwon et al., 2017; Li and Morgan-Kiss, 2019). Eukaryotic phytoplankton dominate the base of the food web and produce organic carbon for a diverse population of heterotrophic bacteria (Kong et al., 2012). In addition to heterotrophic bacteria, there is also evidence of lithoautotroph bacteria in Lakes Bonney and Fryxell (Kong et al., 2012; Dolhi et al., 2015), as well as anoxygenic phototrophs in Lake Fryxell (Karr et al., 2003; Jung et al., 2004). Predatory protists are the apex predators, controlling bacterial and algal prey abundances (Priscu, 1995; Roberts and Laybourn-Parry, 1999; Roberts et al., 2004; Li and Morgan-Kiss, 2019), but other community members such as Chytrid fungi and viruses likely play a role too; although, these taxa are less described (Rojas-Jimenez et al., 2017).

The understanding of viral communities in MDV lake food web and biogeochemical cycling is limited. However, early papers provided some information about the MDV viral morphology, abundance, and productivity (Kepner et al., 1998; Lisle and Priscu, 2004). MDV viral abundances are comparable with temperate waters (10^6^–10^7^ virus like particles per ml), and in many cases viruses even outnumber bacterial cells (Lisle and Priscu, 2004; Säwström et al., 2008). In our lakes of study, viral to bacterial ratios (VBR) found in the east lobe of Lake Bonney are similar to open oceans while the VBR ratio in Lake Fryxell are similar to numbers seen in freshwaters (Maranger and Bird, 1995; Lisle and Priscu, 2004; Säwström et al., 2008). The viral abundances and productivity rates vary between the lakes and throughout the austral summer (Kepner et al., 1998; Lisle and Priscu, 2004). Infection rates have also been estimated using both virus-like particle abundance and mitomycin C induction, respectively, showing that up to 62.5 and 89.5% of total bacteria are infected at any given time (Lisle and Priscu, 2004). These previous studies indicate that viruses in MDV lakes comprise a significant portion of the planktonic community and are actively infecting bacteria; however, their functional roles are still poorly understood.

To further investigate the ecological role of viruses in MDV lakes we examined metagenomes generated from microbial communities residing in the water columns of Lakes Fryxell and Bonney as well as lift-off microbial mats from the edges of Lake Fryxell. The sequenced metagenomes allowed us to identify viral taxonomic distribution among the sites, AMG functional roles, and predict viral hosts. We focus on three main questions, (1) Does the viral taxonomic community differ between lakes (i.e., Lake Fryxell vs. Lake Bonney) and sites (Lakes vs. Microbial Mats)? (2) If AMGs are present, what do they indicate about viral ecological roles in the modulation of host microbe metabolism? (3) What major microbial hosts are being affected by viruses?

## 2 Methods

### 2.1 Site description

The MDVs comprise the largest ice-free area in Antarctica, approximately 4,500 km^2^ in Southern Victoria Land (Levy, 2013). The mean annual temperature is between −15 and − 30°C, with less than 50 mm of precipitation annually (Fountain et al., 1999; Doran et al., 2002). While microbial signatures are found throughout the valleys, life concentrates around liquid water which exists year-round under the permanent ice covers of the lakes or appears in the austral summer when solar radiation is high enough to melt glaciers and snow (Kennedy, 1993; Fountain et al., 1999). Glacial meltwater flows into the lakes for up to 10 weeks a year (McKnight et al., 1999). The permanent ice (3–6 m) that covers MDV lakes prevents wind driven mixing and contributes to water column physical and chemical stratification (Spigel and Priscu, 1998; Li et al., 2016; Kwon et al., 2017). The two lakes in this study, Lakes Fryxell and Bonney, have no outflow, and have different geochemistry and associated biology (Spigel and Priscu, 1998; Takacs and Priscu, 1998; Roberts et al., 2004; Vick-Majors et al., 2014; Kwon et al., 2017; Li and Morgan-Kiss, 2019). With respect to nutrient status, Lake Bonney is more oligotrophic than Lake Fryxell; Lake Bonney is phosphorus deficient whereas Lake Fryxell is nitrogen limited (Priscu, 1995; Dore and Priscu, 2001; Teufel et al., 2017).

The lakes in the MDVs are home to diverse benthic microbial mats, which can detach from the sediments due to production of gas bubbles (Parker et al., 1982; Moorhead et al., 1999). Once detached these microbial mats float to the underside of the surface ice layer where they are carried through the ice by freezing from below and ablation at the surface (Parker et al., 1982). Autochthonous particulate organic matter and dissolved organic matter generated by microbial communities found within the benthic mats play an important role in the primary production of these freshwater ecosystems (Hawes et al., 2005).

### 2.2 Sample collection and sequencing

Microbial communities were sampled in December 2014 from two perennially ice-covered lakes in Taylor Valley, Victoria Land, Antarctica. Triplicate lake water samples were collected from the chemoclines of Lake Fryxell (−77.61034, 163.14271, 9 m depth) and the east lobe of Lake Bonney (−77.71368, 162.44130, 15 m depth) (Tallada et al., 2022) and were collected in 5 separate 1 liter cubitainers that were pre-washed with 10% HCL (Tallada et al., 2022). Water samples were filtered onto 47 mm Pall Supor^®^ 450 polyethersulfone membranes (0.45 μm pore size; Pall Corporation, NY, United States) (Tallada et al., 2022). Six separate desiccated microbial lift-off mat samples were collected from the surface of Lake Fryxell within the GPS area of −77.60491, 163.16315; −77.60473, 163.16290; −77.60463, 163.16405; −77.60495, 163.16495 (Tallada et al., 2022). All samples were collected with alcohol-sterilized forceps and stored at −20°C for 4 weeks and then −80°C for long term storage (Tallada et al., 2022). This study focuses on the viruses present within microbial cells which represent the ecologically relevant viruses at the time of sampling. The selected depths have average bacteria cell width sizes of 0.5 microns, though many diverse free floating viruses can also be caught on 0.45 micron filters.

Shotgun metagenomic library construction and sequencing was performed by the Department of Energy (DOE) Joint Genome Institute (Community Science Program award 1936) using standard protocols on the Illumina HiSeq 2,500 platform (Tallada et al., 2022). The raw sequencing reads were quality controlled using JGI standard protocols and further processed using Trimmomatic (Bolger et al., 2014) to remove adapters and low-quality sequences (Tallada et al., 2022). Sequence read files used in this study are available at NCBI under accession numbers SRP104818, SRP104821, SRP098041, SRP098044, SRP104822, SRP098050, SRP104819, SRP104820, and SRP104823. We excluded the samples MAT-04, MAT-05, MAT-06 from this analysis due to low viral read counts. A table of accession numbers and samples used in this study can be found in Supplementary Table S2.

### 2.3 Viral assembly and annotation

Viral sequences were identified in the JGI quality controlled forward and reverse reads from each sample following a viromics pipeline modified from (Comeau et al., 2017). Briefly, to accurately assess viral communities, metagenomic reads were cross-assembled using the assembly module in the MetaWRAP pipeline, using MEGAHIT version 1.2.9 with a -klist of 21, 29, 39, 59, 79, 119, 141 (Uritskiy et al., 2018). Cross-assembled contigs greater than 2,500 and 5,000 bp were used to identify potential viral contigs and genomes using VirFinder v1.0.0 and VirSorter v.2 (Cyverse) respectively (Roux et al., 2015; Ren et al., 2017). Viral contigs from VirFinder with a q-value of less than 0.1 were retained to minimize the false discovery rate. We kept viral and proviral contigs in categories 1–3 from VirSorter on cyverse which corresponds to the confidence of predicted viruses, i.e., (1) “most confident,” (2) “likely” (3) “possible” (Roux et al., 2015). Redundancies found in identified viral contigs from VirFinder and VirSorter were de-replicated using cd-hit and a sequence similarity threshold of 1.0 (Fu et al., 2012). De-replicated reads were mapped using Bowtie 2 v2.3.5.1 (Langmead and Salzberg, 2012) with the sensitive-local setting. Coverage depths of viral scaffolds were calculated using samtools v0.1.19 (Li et al., 2009) and the jgi_summarize_contig_depths function from MetaBAT 2 v2.12.1 (Kang et al., 2019). Finally, viral contigs with less than 70% coverage were excluded from all results.

### 2.4 Viral diversity metrics

Viral taxonomic annotation was performed with vConTACT2 (v.5) which creates gene sharing networks against the “ProkaryoticViralRefSeq201-Merged” database with DIAMOND and ClusterONE to give putative genus level assignments both within and outside the database (Nepusz et al., 2012; Buchfink et al., 2015; Bin Jang et al., 2019). We compared richness and diversity of viral communities by first minimizing the effects of sequencing depth and library size. To normalize samples, all reads were repeatedly rarefied (*n* = 1,000) according to the smallest library size (*n* = 8,900) using the “phyloseq_mult_raref” function from the metagMisc package in R. Alpha diversity of communities was determined using the inverse Shannon index and richness as the total number of genus clusters. Differences in viral communities between lakes and lift-off mats were visualized using Principal Coordinates Analysis (PCoA, using Bray-Curtis distance) and tested with permutational multivariate analyses of variance (PERMANOVA) in R.

### 2.5 Auxiliary metabolic genes

We extracted and categorized AMGs using DRAM-v (Shaffer et al., 2020). Based on the output of DRAM-v only high confidence AMGs in categories 1 and 2 were retained which are AMGs flanked on one or both sides by viral hallmark genes. To obtain differential abundances of AMGs we paired AMG identifications with their corresponding viral contig abundances.

### 2.6 Host binning, quantification, and taxonomy

We binned our cross-assembly using MetaBat2 v 2.12.1 from the binning function in the MetaWrap pipeline (Uritskiy et al., 2018; Kang et al., 2019). Bacterial bins that were less than 50% complete and over 10% contaminated according to CheckM were excluded from the analysis (Parks et al., 2015). We retained eukaryotic and archaeal bins with high taxonomic assignment from CAT/BAT (von Meijenfeldt et al., 2019). Bins were quantified using the quant_bins module in Metawrap. Taxonomic annotation was performed by CAT/BAT v5.0.3 (von Meijenfeldt et al., 2019). Suggestive assignments and lineage scores from CAT/BAT below 0.9 were trimmed from the predicted host analysis (von Meijenfeldt et al., 2019).

### 2.7 Host predictions

Viral host predictions were made against the bacterial and eukaryal bins using the Phage-Host Interaction Search Tool (PHIST) which predicts viral hosts based on the number of exact shared k-mer matches between viral and host sequences (Zielezinski et al., 2022). Host predictions with significant *p*-values (<0.05) were retained. We performed Procrustes rotation and permutation using the PROTEST function in vegan to search for significant coupling between the viral and bacterial communities.

## 3 Results and discussion

Viruses are major contributors and regulators of ecosystem health and function across the planet. Early studies in the MDV lakes have shown that the abundance of viruses and their prokaryotic infection rates indicates that viruses have ecologically significant roles in these aquatic ecosystems (Kepner et al., 1998; Lisle and Priscu, 2004; Säwström et al., 2008). We analyzed the metagenomes of microbial communities from Lakes Bonney and Fryxell to explore the viral diversity, function and potential community associations from a unique microbially dominated ecosystem where the impact of viral interactions is expected to be especially important.

### 3.1 MDV Lake planktonic and microbial mat viral diversity and composition

**Figure 1:**
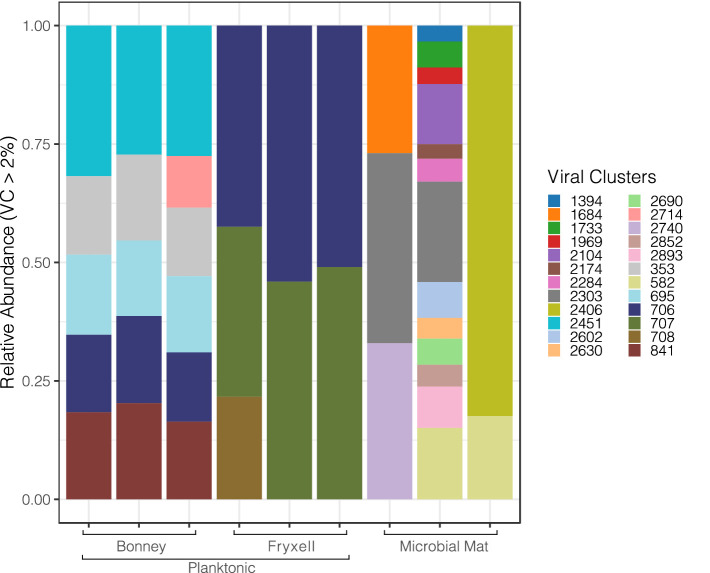
Diversity of major viral clusters in the east lobe of Lake Bonney, Lake Fryxell and the microbial liftoff mats. The counts are based on the abundance of contigs from viral clusters.

Our analysis pipeline recovered 66,376 unique putative viral contigs; we removed singleton contigs that were not mapped to the ProkaryoticViralRefSeq201-Merged database or clustered with each other through vConTACT2. Figure 1 displays the major viral genus clusters comprising >2% of each sample detected in the metagenomic data and reveals the variation among the sample types, sites and replicate samples. Viral cluster composition among the replicate samples was relatively uniform among the planktonic samples but was highly variable across the individual lift-off mat samples. Lift-off mat bacterial communities in MDV lakes are more diverse than plankton samples (Takacs-Vesbach et al., 2010) and similar mats in nearby streams significantly differ at the local spatial scale (Van Horn et al., 2016). Despite differences among replicate samples, Shannon diversity of the viral communities was greatest in the planktonic samples (Lake Fryxell 6.7, Lake Bonney 5.64, lift-off mat 5.61). Viral communities differed significantly among the lakes (Supplementary Figure S1, PERMANOVA, *F* = 9.1349, *R^2^* = 0.75278, *p* = 0.003) and the site (Supplementary Figure S1, lakes vs. lift-off mats; PERMANOVA, *F* = 5.0261, *R^2^* = 0.41793, *p* = 0.02). These findings that viral community structure is distinct between Lakes Bonney and Fryxell agrees with recent studies that showed a strong influence of lake physiochemistry on both bacterial and eukaryal communities (Kwon et al., 2017; Li and Morgan-Kiss, 2019). Viral abundances and productivity also vary between the lakes and depths as shown in other previous studies (Kepner et al., 1998; Lisle and Priscu, 2004). Thus given the range of geochemical variation found in MDV lakes, the viral diversity and its effect on host community function is likely much greater than revealed by this initial viromic analysis. Between the lake samples, the only major viral genus cluster shared among the samples was *706* which was assigned to the *Siphoviridae* family (order *Caudovirales*). The lift-off mats were comprised of a more diverse group of dominant viral clusters than the lake samples and had the most variation amongst replicates. The metadata for the mat samples in this project is limited. However, we know from (Van Horn et al., 2016) that there is tremendous spatial variation in the community composition of Dry Valley stream microbial mats and given that the exact location of where these samples were collected is unknown we can say little about the types of mats collected. Further research into viral communities in microbial mats of the MDVs is required to better understand their community composition and relationship with biotic and abiotic factors.

**Figure 2:**
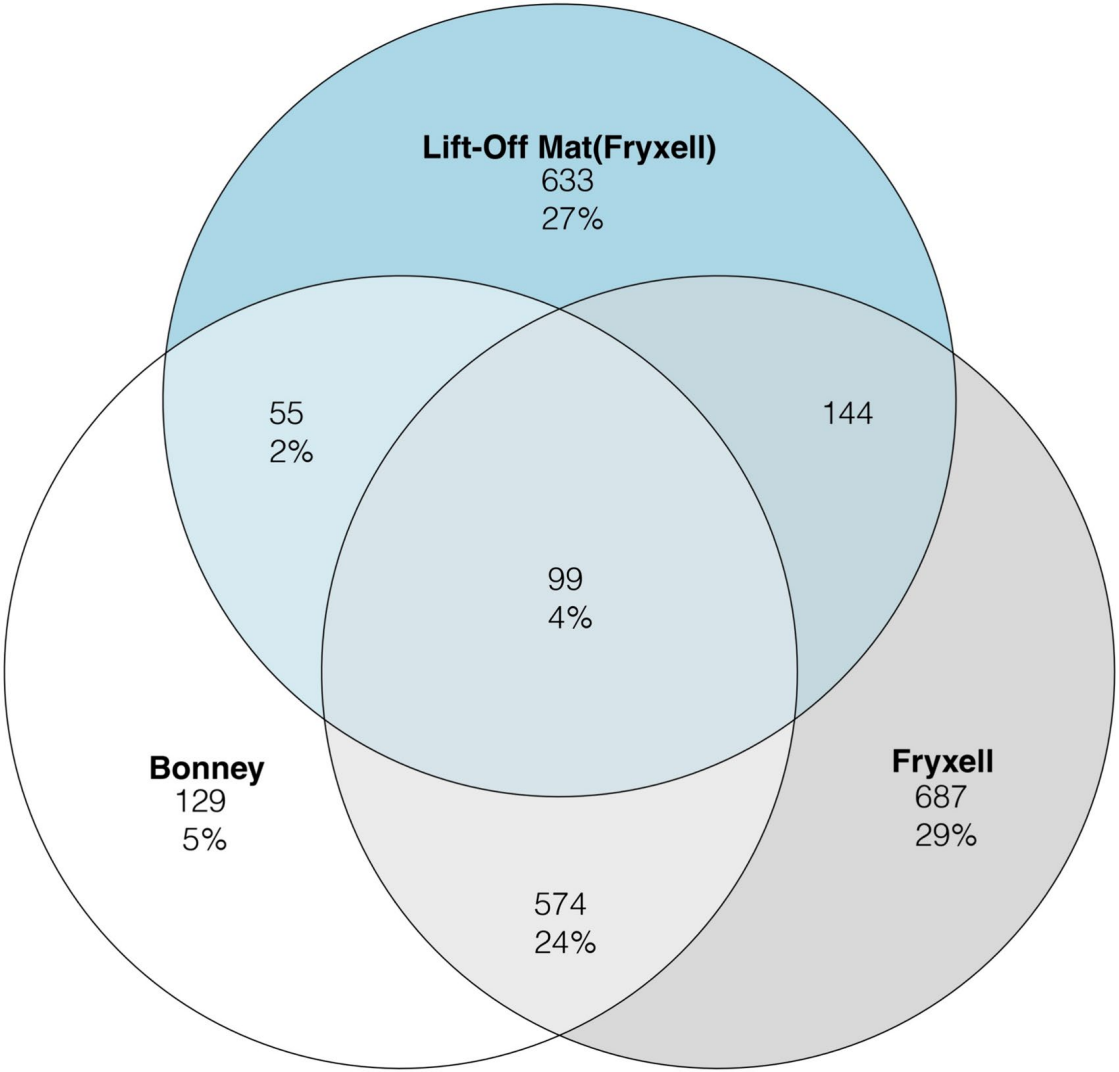
Venn diagram representing the three sample types for the rarefied viral clusters defined by vConTACT2. The number of clusters per sample is displayed in the circle while the number in percentage represents the proportion of all clusters.

To assess viral community distribution amongst the samples we plotted the viral clusters generated from VconTACT2 into Venn diagrams (Figure 2). Viral partitioning among the samples was highly segregated between lakes and sites. In total, only 4% of viral clusters were shared among the three sites. Lake Fryxell had the highest percentage (29%) of unique viral clusters, followed by the lift-off mats (27%) whereas Lake Bonney had the least (5%). Between the samples, the lake samples shared the most viral clusters (25%) (Figure 2). Lastly, Lake Fryxell shares the most viral clusters with the lift-off Mats at 14% while Lake Bonney shared 9% with the lift-off mats. Viral community composition is driven by both abiotic (UV radiation, nutrient concentrations, etc.) and biotic factors such as host metabolism and diversity (Suttle and Chen, 1992; Lisle and Priscu, 2004; Chow and Suttle, 2015). Our results suggest that shared attributes of the physicochemistry and biology of the water column relative to the mats likely account for the highest proportion of shared viral clusters among the planktonic samples. Despite similarities in physicochemistry and biology, overall host communities are distinct (Supplementary Figure S1) and site-specific differences in the viral clusters could be derived from this distinction in host communities. A higher rate of shared clusters among Lake Fryxell and the lift-off mats is potentially due to chemical and geographical similarities since they reside in the same basin.

### 3.2 McMurdo dry valleys auxiliary metabolic genes

**Figure 3:**
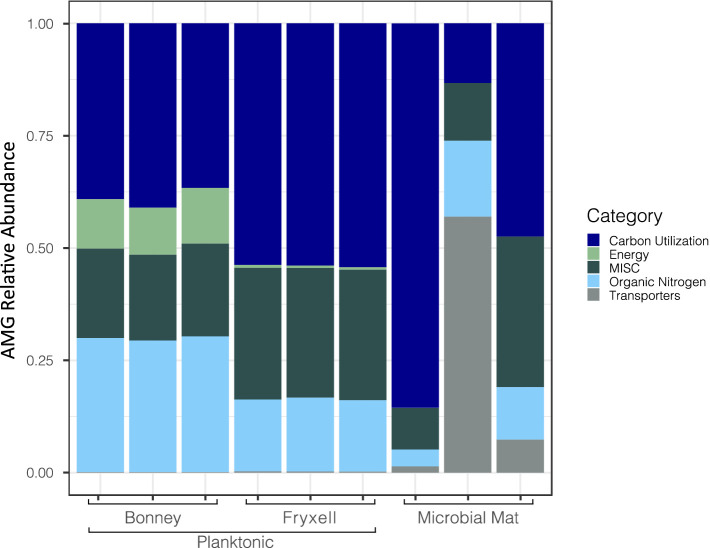
Differences in relative abundances of viral contigs with identified auxiliary metabolic genes across samples. Viral auxiliary metabolic gene categories are defined through DRAM-v.

We searched for putative AMGs using DRAM-v and after quality control 561 AMGs were identified and annotated. Of those found, 68% had no classification in the distilled output generated by DRAM-v; 170 were matched to previously identified AMGs. DRAM-v assigned the AMGs into categories based on the potential function of the putative genes (e.g., energy, carbon utilization, etc.). There were 78 related to carbon utilization, 6 related to energy, 64 miscellaneous, 26 related to organic nitrogen, and 6 related to bacterial transporters (Figure 3). Here, we focused on the genes *pstS, dcm, soxY, and prkB* which are involved in overcoming nutrient and energy limitations (Thompson et al., 2016; Warwick-Dugdale et al., 2019; Heyerhoff et al., 2022). An additional gene *cp12* was discovered in 16 contigs by Virsorter v1.0.0 and could play an important role in viral-mediated carbon cycling because of its role in the Calvin-Benson-Bassham Cycle (Calvin Cycle).

A gene for a phosphate transporter, *pstS* which encodes for a periplasmic high-affinity phosphate-binding protein was detected in putative viral contigs found in Lake Bonney and Lake Fryxell, but was not detected in the lift-off mats. This AMG assists in circumventing phosphorus limitations, which is the primary nutrient limitation in Lake Bonney (Spigel and Priscu, 1998). *pstS* is upregulated in phosphorus deficient environments: increased phosphorus uptake may be required for several processes in viral replication (Gao et al., 2016). We did not detect additional genes involved in phosphorus acquisition such as *phoH* and *phoU*. Increased phosphorus uptake could also synthesize cysteine, which is the only amino acid that can form disulfide bonds that stabilize the viral protein structure (Ashcroft et al., 2005). Viral-mediated phosphorus acquisition could be a significant process in nutrient cycling, particularly in Lake Bonney where the primary limitation is phosphorus (Spigel and Priscu, 1998).

Genetic potential for viral-mediated nitrogen cycling was also detected among the MDV viral AMGs. In all three sample types, methionine degradation genes, specifically DNA (cytosine-5)-methyltransferase (*dcm*) was found. While still being studied, *dcm* may have multiple uses such as aiding in phage capsid stability, helping to circumvent organic nitrogen limitations through methionine degradation, and preventing recognition from prokaryotic methyltransferases (Enav et al., 2014; Heyerhoff et al., 2022; Daniel et al., 2023). The *dcm* gene may enhance the degradation of methionine to then redirect sulfur into the cysteine biosynthesis pathway (Wang et al., 2022). Increased cysteine may allow for stronger capsids and structural stability which could also be useful as an overwinter survival strategy for lytic viruses persisting outside host cells especially, when host abundance such as primary producers, decreases during winter months (Takacs and Priscu, 1998). Additionally, nitrogen is a limiting factor of production and catabolism of amino acids such as methionine and *dcm* could be an additional nitrogen scavenging strategy. Bacteria restriction modification systems remove foreign DNA, but viral methyltrasferases can aid in avoiding host defense systems (Seong et al., 2022). AMGs are expressed during infection which appears to be relatively high in MDV lakes compared with other temperate environments, increasing the importance of AMGs in nutrient limited environments like MDV lakes (Spigel and Priscu, 1998; Lisle and Priscu, 2004; Yau and Seth-Pasricha, 2019). Our data indicates that MDV viruses modulate N-cycling processes in the MDV lakes and microbial mats.

The chemocline samples from Lake Fryxell were the only samples with viral genomes that contained the sulfur oxidation gene *soxY* which encodes part of the thiosulfate oxidizing enzyme complex a key step in the Sox pathway (Jurgensen et al., 2022; Li et al., 2023). MDV lakes contain high concentrations of inorganic and biogenic sulfur and can serve as important energy sources for prokaryotic carbon fixation and potentially overcoming the energy bottleneck of viral replication (Bowman et al., 2016). While incomplete *sox* pathways in bacteria can lead to bottlenecks in the sulfur cycle, it is not uncommon to find incomplete *sox* pathways in viruses because these genes are being used to scavenge energy for nucleotide synthesis (Li et al., 2023). Additionally, viruses may regulate sulfur metabolism differently dependent on environmental and nutritional conditions (Li et al., 2023). In previous bacterial studies, sulfur oxidizing bacteria have been detected in Lake Fryxell and West Lobe Lake Bonney but not in East Lobe Lake Bonney which could explain the absence of sulfur AMGs in ELB (Kong et al., 2012; Dolhi et al., 2015). Phage *soxY* induction may boost sulfur oxidation and lead to prolonged viral infection and replication, increasing burst size (Anantharaman et al., 2014).

Although only detected by VirSorter and not DRAM-v, there is evidence that CP12 serves as an AMG in MDV lake viruses. CP12 is implicated in regulation of photosynthetic processes, specifically, carbon partitioning during the day/night cycle (Tamoi et al., 2005). When expressed during light driven infection, CP12 diminishes phosphoribulokinase (*prkB*) expression (Thompson et al., 2016). The down regulation of the Calvin Cycle and concomitant up-regulation of the Pentose Phosphate Pathway oxidizes carbon stores to produce NADPH which can then be used in nucleotide synthesis. Some contigs that contained CP12 sequences were flanked by phage clusters and accompanied by viral genes within the contig, but not the viral hallmark genes required for annotation by DRAM-v. Manipulation of light driven cycles can increase phage burst size in cyanophage infections, both light and photosynthesis are required for maximal phage production (Amla et al., 1987; Thompson et al., 2016). Both lake metagenomes (but not the microbial mats) indicated the potential for Calvin Cycle inhibition and re-routing the Pentose Phosphate Pathway into nucleotide synthesis through the expression of CP12.

The potential of viral CP12 expression in MDV lake phototrophs could have significant impacts on the carbon cycle in this 24-h daylight ecosystem. Infection by viruses with CP12 could induce downregulation of carbon fixation and increased catabolism of stored carbon, typical of a dark cycle reaction. Additionally, the gene for phosphoribulokinase, PRK, which is traditionally downregulated by the presence of CP12 was detected in four contigs by DRAM-v. Previous studies have shown that PRK has no activity when it exists in the PRK/CP12/GAPDH complex that suppresses the Calvin Cycle (Wedel et al., 1997; Puxty et al., 2016). However, when PRK exists in its dimeric form it is not completely inhibited, even in a dark cycle (Tamoi et al., 2005). Viruses in the MDV lakes may use CP12 and PRK to maximize energy generation by exhausting carbon stores while still converting Ru5P to RuBP to restore the primary substrate for RubisCO in the Calvin Cycle. This potential viral-mediated rewiring of carbon metabolism in the MDV phytoplankton could have significant implications for carbon cycling. Given the high VBR in these lakes, the combination of CP12 and PRK could result in activating dark-driven metabolic processes, despite the 24-light conditions experienced in the austral summer.

### 3.3 Viral host predictions

**Figure 4:**
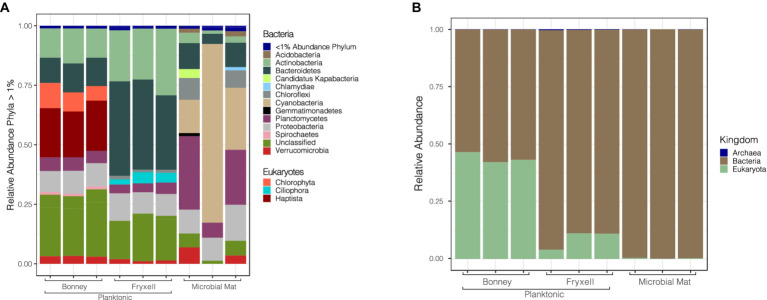
Relative abundance of viruses in conjunction with their putative hosts. **(A)** Predicted viral hosts displayed at the phylum level. Phyla representing less than 1% of the sample are grouped together at the top of the bar plot. **(B)** Predicted viral hosts at the kingdom level.

Viral infection of microbial hosts plays a crucial role in ecosystem dynamics (Suttle, 2005; Knowles et al., 2016; Warwick-Dugdale et al., 2019). We predicted 12,200 multiple (2–17) hosts to 1,367 different retrieved bins for our viral contigs using PHIST. Of those host predictions 773 were predictions of contigs to multiple hosts. Potential hosts included representatives for all three domains of life: Archaea (54), Bacteria (8334), and Eukaryotes (3812). Other studies in nutrient limited environments have also seen a similar diversity of predicted hosts (Cissell and McCoy, 2023; Coutinho et al., 2023). We paired the predicted hosts with their bin abundances to determine host community differences among the samples. Archaeal host predictions were comprised of *Crenarchaeota* (56%), *Euryarchaeota* (40%), and two unclassified hosts (4%). Bacterial host predictions in Lake Bonney were assigned to Actinobacteria (13%), Bacteroidetes (11%), Proteobacteria (9%), whereas Lake Fryxell was dominated by Bacteroidetes (42%), Actinobacteria (22%), and Proteobacteria (10%). Finally, the lift-off mats were dominated by Cyanobacteria (20%), Planctomycetes (25%), and Proteobacteria (9%) (Figure 4).

Archaeal hosts were more frequent in the planktonic samples than the microbial mats but overall were only a small proportion of total predicted hosts. *Crenarchaeota* and *Euryarchaeota* are both phyla that have been previously detected in MDV lakes and contribute to lake biogeochemical cycles (Bowman et al., 2000; Brambilla et al., 2001; Karr et al., 2006). The presence of cyanobacterial hosts in the mats and their absence in the lake samples agrees well with observations that the MDV lakes are generally devoid of planktonic cyanobacteria (Kong et al., 2012; Li and Morgan-Kiss, 2019). Previous studies have shown that bacterial communities in the lakes are dominated by the phylum Bacteroidetes and Actinobacteria (Kwon et al., 2017). The broad range of hosts in our samples shows the potential impact that viruses have on prokaryotic community functionality and highlights the different strategies of viruses in infecting prokaryotes. Host communities with high diversity may elicit a narrow viral infection range due to the increased probability of infecting phylogenetically related members while broad viral host ranges may increase likelihood of exchanging genetic material across populations (Weitz et al., 2013; Hwang et al., 2023; Peter et al., 2024). The majority of our viral predictions have a narrow host range across a broad host diversity, indicative of resource limitations (Weitz et al., 2013). However, the presence of phages infecting multiple hosts does provide evidence that genetic material is moving around microbial communities in MDV lakes via viral infection.

Eukaryotic host predictions in Lake Bonney were much higher relative to the other samples in the study. The eukaryotic sequences in Lake Bonney were dominated by Chlorophyta (12%) and Haptista (22%) (Figure 4) which dominate Lake Bonney and are rarely found in Lake Fryxell (Li and Morgan-Kiss, 2019). The highest abundance among the eukaryotic host predictions belongs to the phylum Haptista. This host is most likely the nanoflagellate *Isochrysis* sp. MDV, which dominates the chemocline of both lobes of Lake Bonney. *Isochrysis* has a number of important functions in the Lake Bonney food web. It is a constitutive mixotroph, utilizing light for energy and supplementing energy and nutrients by phagotrophy (Li and Morgan-Kiss, 2019). During the polar winter when it is dark for 4 months, Haptophytes dominate the algal communities of Lake Bonney, likely because they can switch to heterotrophic metabolism (Patriarche et al., 2021). Furthermore, near the end of polar winter, Haptophyte communities have been observed to rapidly collapse, which has been attributed to predator activity or viral lysis (Patriarche et al., 2021). Our discovery of abundant viruses associated with a dominant algal host suggests that algal viruses likely play an active role in controlling the phytoplankton populations and recycling carbon and nutrients in Lake Bonney. Conversely, we did not detect any evidence of viral hosts for the dominant Cryptophyte communities of Lake Fryxell, potentially because the Lake Fryxell cryptophytes are predated upon by ciliates rather than viruses (Roberts and Laybourn-Parry, 1999).

**Figure 5:**
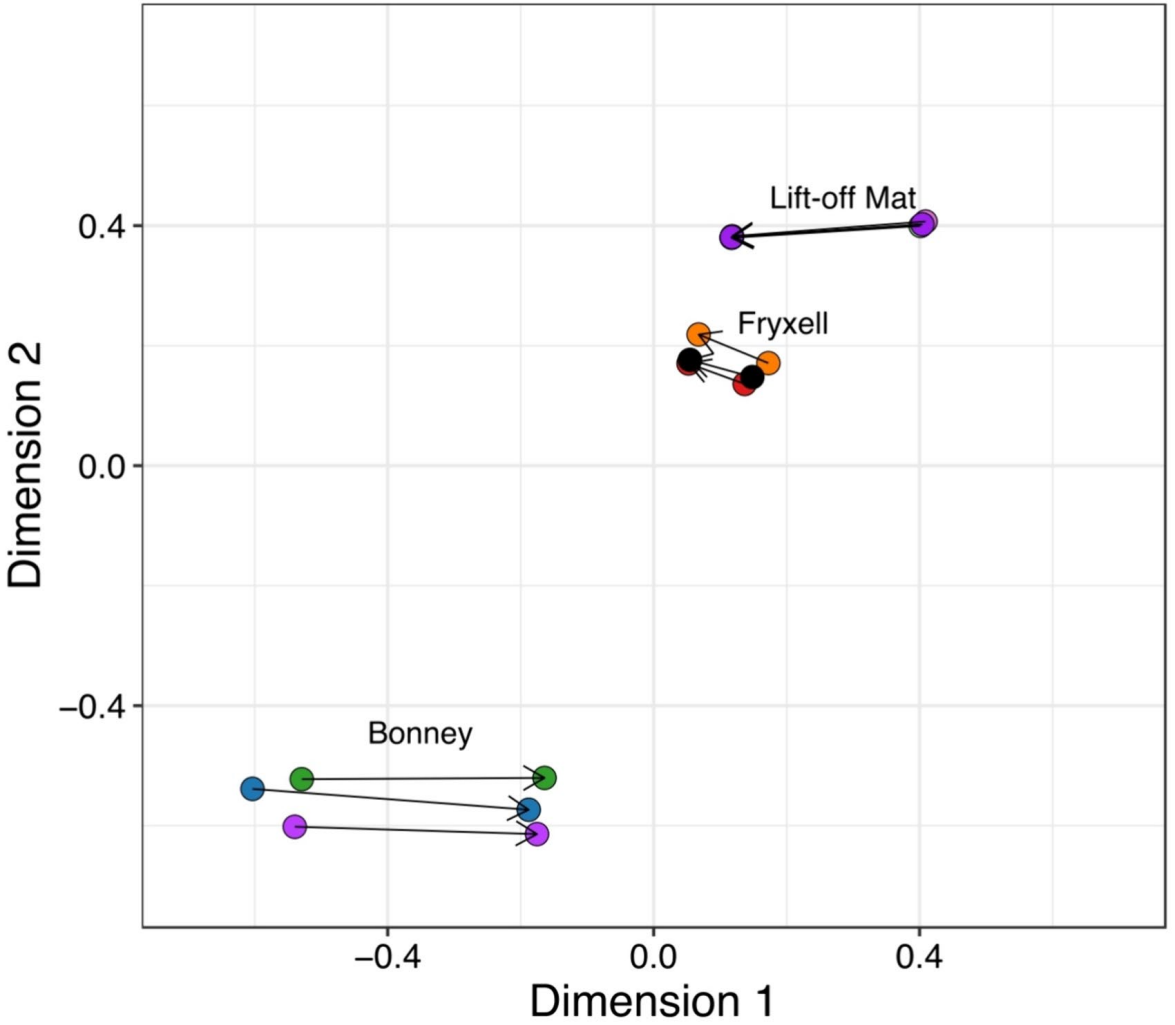
Procrustes rotation and permutation visualizes the coupling between viral and bacterial communities. Using Bray-Curtis distances, bacterial and viral ordinations are rescaled and connected by a line.

We used Procrustes rotation and permutation analysis, to search for significant coupling between the microbial and viral communities. Our analyses show that viral and bacterial community members are tightly linked in the MDVs (Procrustes rotation correlation 0.997, *p* = 0.001) and changes between these communities are coupled (Figure 5). If paired with life-cycle dynamics from previous studies and the host predictions described here, the coupling of the communities may largely be driven by the most abundant members of the bacterial and eukaryotic communities (Thingstad, 2000; Bekliz et al., 2022). Changes in the one community likely contribute to changes in the other community in the lakes in the MDVs.

## 4 Conclusion

To the best of our knowledge, this is the first study in the lakes of the MDVs to use high-throughput metagenomics to examine viral communities and their relationships with their hosts. Previous studies have focused on viral community dynamics using particle counts, microscopy, and calculated rates of lysogeny (Kepner et al., 1998; Lisle and Priscu, 2004). While integral to our current understanding of viruses, previous studies did not reveal the diversity or potential influence of viruses as seen through the virome lens. Our study shows that viruses in the MDVs are diverse and uniquely distributed throughout the MDVs. We also showed that viruses infect the dominant phyla commonly found in the lakes of the MDVs and while infecting their hosts they can hijack host cell metabolism to circumvent the nutrient limitations of their current environments. AMGs likely account for the discrepancy between the viral infection estimates in Lisle and Priscu (2004). Viruses play a dual role in nutrient cycling in the MDVs though lysis and host cell metabolism augmentation. Due to climate change these current viral roles will likely alter as MDV lakes are predicted to be seasonally ice free within this century (Obryk et al., 2019). This study provides the foundation for future viromics work which is key to understanding climate impacts on MDV lakes.

## Data availability statement

The data presented in this study is available in NCBI under SRA accession numbers SRP104818, SRP104821, SRP098041, SRP098044, SRP104822, SRP098050, SRP104819, SRP104820, and SRP104823. The original contributions of this study are presented in the article/Supplementary Material, further inquiries can be directed to the corresponding author.

## Author contributions

DR: Formal analysis, Writing – original draft. RM-K: Conceptualization, Funding acquisition, Writing – review & editing. ZW: Funding acquisition, Methodology, Writing – review & editing. CT-V: Funding acquisition, Supervision, Writing – review & editing.
