# Supplementary material for: Antarctic lake viromes reveal potential virus associated influences on nutrient cycling in ice-covered lakes
Source: Front Microbiol. 2024 Sep 10;15:1422941. doi: 10.3389/fmicb.2024.1422941 (PMC11421388; doi:10.3389/fmicb.2024.1422941)
Supplement: Supplementary file 1 [file Data_Sheet_1.docx]

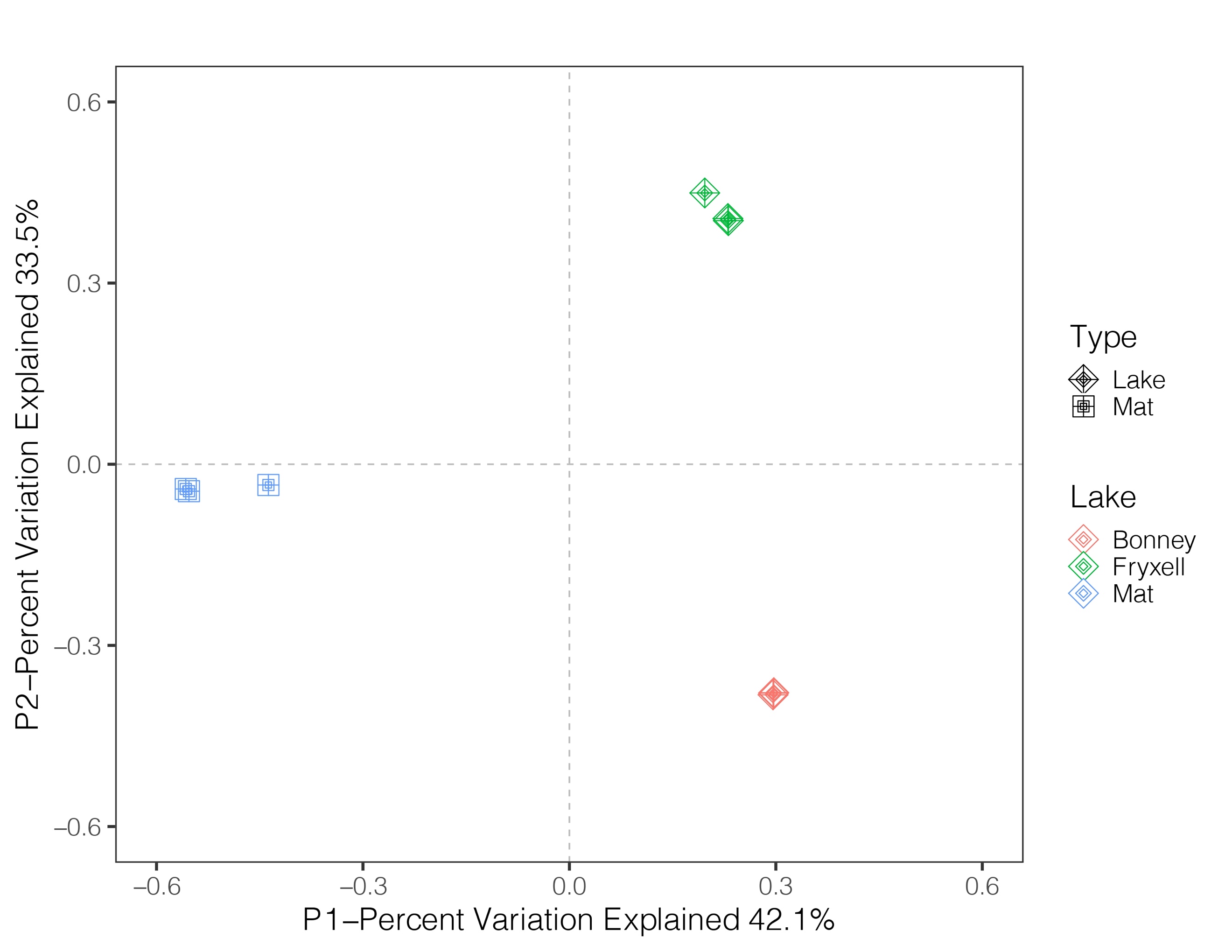


Supplemental Figure 1

Principle coordinates analysis (PCoA) summarizing the beta diversity of viral community composition between Lakes Fryxell and Bonney along with the lift-off mats. PCoA is based on Bray-Curtis differences at the VC level.

Supplemental Figure 2

Sample coverages for metagenomic viral diversity

Supplemental Figure 3

AMG abundances not relativized

Supplemental Figure 4

Viral abundances not relativized

| **Genome** | **Order** | **Family** | **Genus** | **VC** |
| --- | --- | --- | --- | --- |
| Achromobacter~phage~phiAxp-1 | Caudovirales | Siphoviridae | Unassigned | 1_0 |
| Acinetobacter~phage~IMEAB3 | Caudovirales | Siphoviridae | Lokivirus | 1_0 |
| Acinetobacter~phage~Loki | Caudovirales | Siphoviridae | Lokivirus | 1_0 |
| Burkholderia~phage~vB_BceS_KL1 | Caudovirales | Siphoviridae | Septima3virus | 1_0 |
| Paracoccus~phage~vB_PmaS-R3 | Caudovirales | Siphoviridae | Unassigned | 1_0 |
| Pseudomonas~phage~73 | Caudovirales | Siphoviridae | Septima3virus | 1_0 |
| Pseudomonas~phage~PaMx42 | Caudovirales | Siphoviridae | Septimatrevirus | 1_0 |
| Pseudomonas~phage~vB_Pae-Kakheti25 | Caudovirales | Siphoviridae | Septima3virus | 1_0 |
| Pseudomonas~phage~vB_PaeS_SCH_Ab26 | Caudovirales | Siphoviridae | Septima3virus | 1_0 |
| Rhodobacter~phage~RcSpartan | Caudovirales | Siphoviridae | Titanvirus | 1_0 |
| Rhodobacter~phage~RcTitan | Caudovirales | Siphoviridae | Titanvirus | 1_0 |
| Stenotrophomonas~phage~vB_SmaS-DLP_2 | Caudovirales | Siphoviridae | Septimatrevirus | 1_0 |
| VF_VS_62535 | Caudovirales | Siphoviridae | Unassigned | 1_0 |
| Bdellovibrio~phage~phi1422 | Caudovirales | Myoviridae | Unassigned | 124_0 |
| VF_VS_62686 | Caudovirales | Myoviridae | Unassigned | 124_0 |
| VF_VS_62736 | Caudovirales | Myoviridae | Unassigned | 124_0 |
| Salmonella~phage~SEN34 | Caudovirales | Myoviridae | Brunovirus | 126_0 |
| VF_VS_117 | Caudovirales | Myoviridae | Unassigned | 126_0 |
| Vibrio~phage~VBM1 | Caudovirales | Myoviridae | Unassigned | 128_0 |
| VF_VS_127 | Caudovirales | Myoviridae | Unassigned | 128_0 |
| Clavibacter~phage~CMP1 | Caudovirales | Siphoviridae | Cimpunavirus | 192_0 |
| Clavibacter~phage~CN1A | Caudovirales | Siphoviridae | Cinunavirus | 192_0 |
| Microbacterium~phage~vB_MoxS-ISF9 | Caudovirales | Siphoviridae | Unassigned | 192_0 |
| VF_VS_1640 | Caudovirales | Siphoviridae | Unassigned | 192_0 |
| VF_VS_1744 | Caudovirales | Siphoviridae | Unassigned | 192_0 |
| VF_VS_2631 | Caudovirales | Siphoviridae | Unassigned | 192_0 |
| Streptomyces~phage~Bing | Caudovirales | Siphoviridae | Bingvirus | 197_0 |
| Streptomyces~phage~DrGrey | Caudovirales | Siphoviridae | Rimavirus | 197_0 |
| Streptomyces~phage~Rima | Caudovirales | Siphoviridae | Rimavirus | 197_0 |
| Streptomyces~phage~Scap1 | Caudovirales | Siphoviridae | Scapunavirus | 197_0 |
| VF_VS_62 | Caudovirales | Siphoviridae | Unassigned | 197_0 |
| Arthrobacter~phage~Laroye | Caudovirales | Siphoviridae | Laroyevirus | 203_0 |
| VF_VS_10571 | Caudovirales | Siphoviridae | Unassigned | 203_0 |
| VF_VS_1073 | Caudovirales | Siphoviridae | Unassigned | 203_0 |
| VF_VS_3277 | Caudovirales | Siphoviridae | Unassigned | 203_0 |
| VF_VS_4403 | Caudovirales | Siphoviridae | Unassigned | 203_0 |
| VF_VS_63974 | Caudovirales | Siphoviridae | Unassigned | 203_0 |
| VF_VS_886 | Caudovirales | Siphoviridae | Unassigned | 203_0 |
| Azospirillum~phage~Cd | Caudovirales | Siphoviridae | Unassigned | 215_0 |
| VF_VS_66258 | Caudovirales | Siphoviridae | Unassigned | 215_0 |
| Achromobacter~phage~JWF | Caudovirales | Siphoviridae | Unassigned | 22_0 |
| Caulobacter~phage~Seuss | Caudovirales | Siphoviridae | Seussvirus | 22_0 |
| VF_VS_564 | Caudovirales | Siphoviridae | Unassigned | 22_0 |
| Streptomyces~phage~BRock | Caudovirales | Myoviridae | Unassigned | 227_0 |
| VF_VS_251 | Caudovirales | Myoviridae | Unassigned | 227_0 |
| VF_VS_265 | Caudovirales | Myoviridae | Unassigned | 227_0 |
| VF_VS_62689 | Caudovirales | Myoviridae | Unassigned | 227_0 |
| VF_VS_62808 | Caudovirales | Myoviridae | Unassigned | 227_0 |
| Xylella~phage~Xfas53 | Caudovirales | Podoviridae | Unassigned | 275_0 |
| VF_VS_2729 | Caudovirales | Podoviridae | Unassigned | 275_0 |
| VF_VS_477 | Caudovirales | Podoviridae | Unassigned | 275_0 |
| VF_VS_62940 | Caudovirales | Podoviridae | Unassigned | 275_0 |
| VF_VS_63338 | Caudovirales | Podoviridae | Unassigned | 275_0 |
| VF_VS_146 | Caudovirales | Podoviridae | Unassigned | 275_0 |
| Brucella~phage~BiPBO1 | Caudovirales | Siphoviridae | Unassigned | 287_0 |
| Rhizobium~phage~16-3 | Caudovirales | Siphoviridae | Unassigned | 287_0 |
| VF_VS_1132 | Caudovirales | Siphoviridae | Unassigned | 287_0 |
| VF_VS_193 | Caudovirales | Siphoviridae | Unassigned | 287_0 |
| VF_VS_62738 | Caudovirales | Siphoviridae | Unassigned | 287_0 |
| VF_VS_63018 | Caudovirales | Siphoviridae | Unassigned | 287_0 |
| Myxococcus~phage~Mx8 | Caudovirales | Podoviridae | Myxoctovirus | 294_0 |
| VF_VS_1043 | Caudovirales | Podoviridae | Myxoctovirus | 294_0 |
| VF_VS_579 | Caudovirales | Podoviridae | Myxoctovirus | 294_0 |
| VF_VS_62792 | Caudovirales | Podoviridae | Myxoctovirus | 294_0 |
| VF_VS_62828 | Caudovirales | Podoviridae | Myxoctovirus | 294_0 |
| VF_VS_62856 | Caudovirales | Podoviridae | Myxoctovirus | 294_0 |
| VF_VS_62865 | Caudovirales | Podoviridae | Myxoctovirus | 294_0 |
| VF_VS_63010 | Caudovirales | Podoviridae | Myxoctovirus | 294_0 |
| Alteromonas~phage~vB_AcoS-R7M | Unassigned | Unassigned | Unassigned | 3_0 |
| Pantoea~phage~vB_PagS_Vid5 | Caudovirales | Siphoviridae | Vidquintavirus | 3_0 |
| Pseudomonas~phage~NP1 | Caudovirales | Siphoviridae | Np1virus | 3_0 |
| Pseudomonas~phage~PaMx25 | Caudovirales | Siphoviridae | Np1virus | 3_0 |
| VF_VS_45 | Caudovirales | Siphoviridae | Unassigned | 3_0 |
| Burkholderia~phage~BcepB1A | Caudovirales | Myoviridae | Unassigned | 306_0 |
| Shewanella~sp.~phage~1/41 | Caudovirales | Myoviridae | Unassigned | 306_0 |
| VF_VS_4063 | Caudovirales | Myoviridae | Unassigned | 306_0 |
| VF_VS_62669 | Caudovirales | Myoviridae | Unassigned | 306_0 |
| VF_VS_62798 | Caudovirales | Myoviridae | Unassigned | 306_0 |
| Pelagibacter~phage~HTVC010P | Caudovirales | Podoviridae | Unassigned | 324_0 |
| VF_VS_12731 | Caudovirales | Podoviridae | Unassigned | 324_0 |
| VF_VS_1745 | Caudovirales | Podoviridae | Unassigned | 324_0 |
| VF_VS_1979 | Caudovirales | Podoviridae | Unassigned | 324_0 |
| VF_VS_2047 | Caudovirales | Podoviridae | Unassigned | 324_0 |
| VF_VS_62615 | Caudovirales | Podoviridae | Unassigned | 324_0 |
| VF_VS_62656 | Caudovirales | Podoviridae | Unassigned | 324_0 |
| VF_VS_62708 | Caudovirales | Podoviridae | Unassigned | 324_0 |
| VF_VS_62822 | Caudovirales | Podoviridae | Unassigned | 324_0 |
| VF_VS_62842 | Caudovirales | Podoviridae | Unassigned | 324_0 |
| VF_VS_66266 | Caudovirales | Podoviridae | Unassigned | 324_0 |
| VF_VS_8066 | Caudovirales | Podoviridae | Unassigned | 324_0 |
| Cellulophaga~phage~phi38:1 | Caudovirales | Podoviridae | Unassigned | 353_0 |
| VF_VS_3426 | Caudovirales | Podoviridae | Unassigned | 353_0 |
| VF_VS_480 | Caudovirales | Podoviridae | Unassigned | 353_0 |
| VF_VS_525 | Caudovirales | Podoviridae | Unassigned | 353_0 |
| VF_VS_5504 | Caudovirales | Podoviridae | Unassigned | 353_0 |
| VF_VS_611 | Caudovirales | Podoviridae | Unassigned | 353_0 |
| VF_VS_85 | Caudovirales | Podoviridae | Unassigned | 353_0 |
| VF_VS_990 | Caudovirales | Podoviridae | Unassigned | 353_0 |
| Cellulophaga~phage~phi46:1 | Caudovirales | Siphoviridae | Unassigned | 365_0 |
| VF_VS_470 | Caudovirales | Siphoviridae | Unassigned | 365_0 |
| Pseudomonas~phage~PMBT14 | Caudovirales | Siphoviridae | Unassigned | 4_0 |
| VF_VS_16204 | Caudovirales | Siphoviridae | Unassigned | 4_0 |
| VF_VS_63086 | Caudovirales | Siphoviridae | Unassigned | 4_0 |
| VF_VS_63180 | Caudovirales | Siphoviridae | Unassigned | 4_0 |
| Xanthomonas~phage~Xp15 | Caudovirales | Siphoviridae | Unassigned | 428_0 |
| VF_VS_62483 | Caudovirales | Siphoviridae | Unassigned | 428_0 |
| VF_VS_62373 | Caudovirales | Siphoviridae | Unassigned | 428_0 |
| Ralstonia~phage~RSK1 | Caudovirales | Podoviridae | Unassigned | 445_0 |
| VF_VS_62581 | Caudovirales | Podoviridae | Unassigned | 445_0 |
| Faecalibacterium~phage~FP_Brigit | Caudovirales | Myoviridae | Brigitvirus | 461_0 |
| VF_VS_62407 | Caudovirales | Myoviridae | Unassigned | 461_0 |
| VF_VS_62513 | Caudovirales | Myoviridae | Unassigned | 461_0 |
| VF_VS_62647 | Caudovirales | Myoviridae | Unassigned | 461_0 |
| VF_VS_62675 | Caudovirales | Myoviridae | Unassigned | 461_0 |
| VF_VS_62802 | Caudovirales | Myoviridae | Unassigned | 461_0 |
| VF_VS_62810 | Caudovirales | Myoviridae | Unassigned | 461_0 |
| VF_VS_63066 | Caudovirales | Myoviridae | Unassigned | 461_0 |
| Rhodovulum~phage~vB_RhkS_P1 | Caudovirales | Siphoviridae | Unassigned | 462_0 |
| VF_VS_62627 | Caudovirales | Siphoviridae | Unassigned | 462_0 |
| VF_VS_62818 | Caudovirales | Siphoviridae | Unassigned | 462_0 |
| uncultured~crAssphage | Caudovirales | Podoviridae | Unassigned | 469_0 |
| VF_VS_13 | Caudovirales | Podoviridae | Unassigned | 469_0 |
| Gordonia~phage~GMA1 | Caudovirales | Siphoviridae | Unassigned | 484_0 |
| Rhodococcus~phage~Jace | Caudovirales | Siphoviridae | Unassigned | 484_0 |
| Rhodococcus~phage~REQ3 | Caudovirales | Siphoviridae | Unassigned | 484_0 |
| VF_VS_62367 | Caudovirales | Siphoviridae | Unassigned | 484_0 |
| Gordonia~phage~GMA6 | Caudovirales | Myoviridae | Bendigovirus | 499_0 |
| VF_VS_382 | Caudovirales | Myoviridae | Unassigned | 499_0 |
| Puniceispirillum~phage~HMO-2011 | Caudovirales | Podoviridae | Unassigned | 536_0 |
| VF_VS_1104 | Caudovirales | Podoviridae | Unassigned | 536_0 |
| VF_VS_12411 | Caudovirales | Podoviridae | Unassigned | 536_0 |
| VF_VS_12724 | Caudovirales | Podoviridae | Unassigned | 536_0 |
| VF_VS_2063 | Caudovirales | Podoviridae | Unassigned | 536_0 |
| VF_VS_4642 | Caudovirales | Podoviridae | Unassigned | 536_0 |
| Planktothrix~phage~PaV-LD | Caudovirales | Siphoviridae | Unassigned | 558_0 |
| VF_VS_62695 | Caudovirales | Siphoviridae | Unassigned | 558_0 |
| Thalassomonas~phage~BA3 | Caudovirales | Podoviridae | Unassigned | 570_0 |
| VF_VS_12438 | Caudovirales | Podoviridae | Unassigned | 570_0 |
| VF_VS_1384 | Caudovirales | Podoviridae | Unassigned | 570_0 |
| VF_VS_328 | Caudovirales | Podoviridae | Unassigned | 570_0 |
| VF_VS_426 | Caudovirales | Podoviridae | Unassigned | 570_0 |
| VF_VS_482 | Caudovirales | Podoviridae | Unassigned | 570_0 |
| VF_VS_585 | Caudovirales | Podoviridae | Unassigned | 570_0 |
| VF_VS_6224 | Caudovirales | Podoviridae | Unassigned | 570_0 |
| VF_VS_62559 | Caudovirales | Podoviridae | Unassigned | 570_0 |
| VF_VS_62719 | Caudovirales | Podoviridae | Unassigned | 570_0 |
| VF_VS_62785 | Caudovirales | Podoviridae | Unassigned | 570_0 |
| VF_VS_62819 | Caudovirales | Podoviridae | Unassigned | 570_0 |
| VF_VS_63202 | Caudovirales | Podoviridae | Unassigned | 570_0 |
| VF_VS_7548 | Caudovirales | Podoviridae | Unassigned | 570_0 |
| VF_VS_7775 | Caudovirales | Podoviridae | Unassigned | 570_0 |
| VF_VS_7900 | Caudovirales | Podoviridae | Unassigned | 570_0 |
| VF_VS_963 | Caudovirales | Podoviridae | Unassigned | 570_0 |
| Vibrio~phage~VvAW1 | Caudovirales | Podoviridae | Unassigned | 570_0 |
| Pelagibacter~phage~HTVC008M | Caudovirales | Myoviridae | Unassigned | 578_0 |
| VF_VS_6 | Caudovirales | Myoviridae | Unassigned | 578_1 |
| Caulobacter~phage~Cr30 | Caudovirales | Myoviridae | Unassigned | 578_2 |
| Pseudomonas~phage~pf16 | Caudovirales | Myoviridae | Chakrabartyvirus | 578_3 |
| Acidovorax~phage~ACP17 | Caudovirales | Myoviridae | Busanvirus | 578_4 |
| Phormidium~phage~MIS-PhV1A | Unassigned | Unassigned | Unassigned | 582_0 |
| Phormidium~phage~MIS-PhV1B | Unassigned | Unassigned | Unassigned | 582_0 |
| VF_VS_63753 | Unassigned | Unassigned | Unassigned | 582_0 |
| VF_VS_63963 | Unassigned | Unassigned | Unassigned | 582_0 |
| VF_VS_64415 | Unassigned | Unassigned | Unassigned | 582_0 |
| Riemerella~phage~RAP44 | Caudovirales | Siphoviridae | Unassigned | 635_0 |
| VF_VS_62439 | Caudovirales | Siphoviridae | Unassigned | 635_0 |
| Stenotrophomonas~phage~SMA9 | Tubulavirales | Inoviridae | Staminivirus | 649_0 |
| VF_VS_7280 | Tubulavirales | Inoviridae | Unassigned | 649_0 |
| Nitrincola~phage~1M3-16 | Caudovirales | Unassigned | Unassigned | 67_0 |
| VF_VS_200 | Caudovirales | Unassigned | Unassigned | 67_0 |

Supplementary Table 1

vConTACT2 taxonomy cluster classification of all contigs able to be identified by the RefSeq 201 database.

Supplementary Table 2

| Accession numbers on SRA | Sample IDs |
| --- | --- |
| SRP104818 | MAT-01 |
| SRP104821 | MAT-02 |
| SRP098041 | MAT-03 |
| SRP098044 | FRX-01 |
| SRP104822 | FRX-02 |
| SRP098050 | FRX-03 |
| SRP104819 | BON-01 |
| SRP104820 | BON-02 |
| SRP104823 | BON-03 |
